# Ideal Oscillation of a Hydrogenated Deformable Rotor in a Gigahertz Rotation–Translation Nanoconverter at Low Temperatures

**DOI:** 10.3390/s20071969

**Published:** 2020-04-01

**Authors:** Bo Song, Jiao Shi, Jinbao Wang, Jianhu Shen, Kun Cai

**Affiliations:** 1 College of Water Resources and Architectural Engineering, Northwest A&F University, Yangling 712100, China; songbo943@163.com (B.S.); shijiaoau@163.com (J.S.); 2 State Key Laboratory of Structural Analysis for Industrial Equipment, Dalian University of Technology, Dalian 116024, China; 3 School of Port and Transportation Engineering, Zhejiang Ocean University, Zhoushan 316022, China; jinbaow@zjou.edu.cn; 4 Centre for Innovative Structures and Materials, School of Engineering, RMIT University, Melbourne, VIC 3001, Australia; jianhu.shen@rmit.edu.au

**Keywords:** nano-oscillator, rotation–translation converter, nanodevice, molecular dynamics, hydrogenation

## Abstract

It was discovered that large-amplitude axial oscillation can occur on a rotor with an internally hydrogenated deformable part (HDP) in a rotation–translation nanoconverter. The dynamic outputs of the system were investigated using molecular dynamics simulations. When an input rotational frequency (100 GHz > *ω* > 20 GHz) was applied at one end of the rotor, the HDP deformed under the centrifugal and van der Waals forces, which simultaneously led to the axial translation of the other end of the rotor. Except at too high an input rotational frequency (e.g., >100 GHz), which led to eccentric rotation and even collapse of the system, the present system could generate a periodic axial oscillation with an amplitude above 0.5 nm at a temperature below 50 K. In other ranges of temperature and amplitude, the oscillation dampened quickly due to the drastic thermal vibrations of the atoms. Furthermore, the effects of the hydrogenation scheme and the length of HDP on the equilibrium position, amplitude, and frequency of oscillation were investigated. The conclusions can be applied to the design of an ideal nano-oscillator based on the present rotation–translation converter model.

## 1. Introduction

Due to the extremely high in-plane/in-shell strength [1,2] and ideal inter-layer superlubrication [3,4,5], pure *sp^2^* carbon materials, such as carbon nanotubes (CNTs) [6] and graphene [7], are widely adopted in the design and fabrication of nanodevices, such as nanomotors [8,9], nanobearings [3,10], nano-oscillators [11,12,13], and nanopumps [14]. Meanwhile, some of them have already been realized in nanofabrication. To ensure those nanodevices work normally, numerous driving methods, such as a thermally driven strategy [15,16], electrical strategy [9,17], and fluid strategy [18,19], have been proposed. 

In 2000, several experiments [3,20,21] demonstrated the low interaction between adjacent walls of multi-walled CNTs (MWCNTs) by pulling the inner tubes out. Since then, extensive attention has been paid to CNT-based nano-oscillators [12,22,23,24,25,26]. Zheng and Jiang [11] initially proposed the model of an axial gigahertz oscillator from MWCNTs. Legoas et al. [27] presented a molecular dynamics study of nano-oscillation with respect to dynamical aspects, such as temperature, force, and energy temporal fluctuations. However, even though the friction between two concentric CNTs is very low, the oscillation amplitude still dampens quickly [28,29]. How to generate a stable and sustainable oscillation is the major challenge for the practical application of an oscillator. Many researchers have tried to overcome the damping oscillation by introducing an external driven field [30,31], but this will interfere or even cover the original oscillation frequencies of the oscillator.

To address this challenge, in 2014, Cai et al. [32] found that in an NVE (NVE: constant Number of atoms, constant Volume and Energy of system) ensemble, long-life stable oscillations can be generated by introducing a high-speed rotating rotor in the stator. However, an NVE ensemble is not as easily realized as an NVT (NVT: constant Number of atoms, constant Volume and Temperature of system) ensemble in a laboratory. When studying the dynamic response of a nanomotor made from triple-walled CNTs, Shi et al. [33] found the concurrence of oscillations and rotation of the rotors in the fixed outer tubes when the system was in an NVT ensemble. Cai et al. [34] further discussed the effects of the length of the inner rotor, the temperature, and the input rotational frequency of the middle rotor on the dynamic response of the inner rotor, and noticed that the inner rotor could oscillate periodically with a stable amplitude, especially at relatively low temperature. 

Different from the above works, Song et al. [35] presented a nanodevice called a rotation–translation system in an NVT ensemble. In their model, a deformable part (DP) made of graphene was connected to the CNT rotor. The DP deforms when the rotation-induced centrifugal force [36] is strong enough, and the axial translation of the rotor happens simultaneously. Unfortunately, DP may be self-bonded into a narrow ring under high rotational speed, which is unrecoverable and unstable. To solve this problem, Song et al. [37] hydrogenated the DP properly [38,39,40,41]. They found that the dynamic responses of DP and hydrogenated DP (HDP) at room temperature were very different, and proved the recoverability of the HDP. However, these two works could not generate ideal oscillation with a large amplitude. 

For a nanosystem that is placed in an NVT ensemble, random thermal vibration is intrinsic for atoms. Hence, temperature plays a critical role in the dynamic response and output signal of the system [34,42,43,44]. In general, a higher temperature means drastic thermal vibrations and more friction between adjacent walls of the MWCNTs. At a low temperature, the thermal vibration is weak and some novel phenomena may appear [34]. 

In the present work, a rotor with a recoverable HDP was placed in an NVT ensemble with a temperature under 150 K. The dynamic response of the system and the axial motion of the output end of the rotor were investigated via molecular dynamics simulations. Our results have great potential for the design of various nanodevices, such as high-speed digital and analog circuits, and memory cells.

## 2. Results and Discussion

### 2.1. Dynamic Behaviors for the Z2H6 System at an Extremely Low Temperature, e.g., 8 K

**Figure 1:**
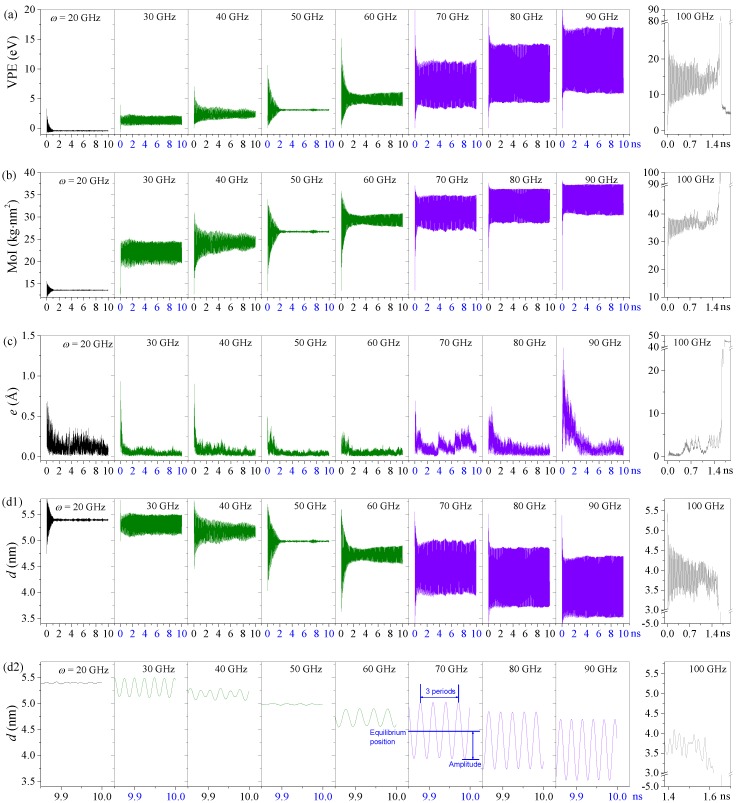
Historical curves of dynamic outputs for the Z2H6 system under different rotation frequencies at 8 K: (**a**) variation of the potential energy (VPE) of the HDP, (**b**) moment of inertia (MoI) of the HDP about the z-axis, (**c**) eccentricity (*e*) of the HDP from the z-axis, (**d1**) the axial translation of the R-rotor, *d*, and (**d2**) the amplified view of *d* during [9.85, 10.00] ns “ZmHn” means n columns of carbon atoms on HDP with an axial length of Zm are hydrogenated, e.g., Z2H6 indicates six columns hydrogenated carbon atoms on the HDP with a length of Z2. Details of the HDP are given in Section 3.

When an input rotational frequency was suddenly applied on the rotor, the HDP deformed drastically in the first 2 ns, and therefore led to obvious fluctuations of the variation of potential energy (VPE) curves (Figure 1a). After that, the amplitudes of the fluctuation of the VPE curves reduced and gradually tended toward being stable. In general, the mean value of the VPE of the HDP increased with the input rotational speed (*ω*), and a larger mean value indicates the deformation of the HDP was greater.

**Figure 2:**
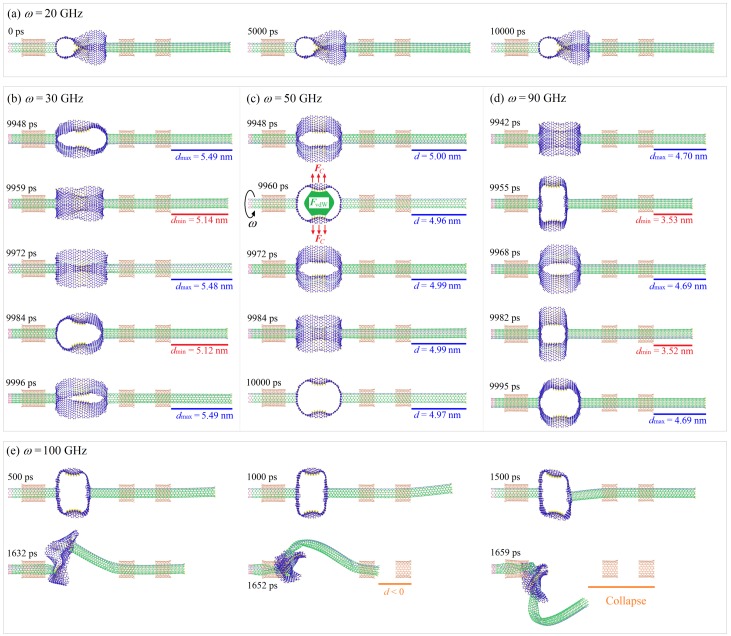
Configurations of the Z2H6 system under different conditions at 8 K: (**a**) *ω* = 20 GHz, (**b**) *ω* = 30 GHz, (**c**) *ω* = 50 GHz, (**d**) *ω* = 90 GHz, and (**e**) *ω* = 100 GHz. In (a), the HDP looked like a “∞,” and the two hydrogenated areas were in contact when *ω* was too low. As shown in (c), the vdW forces led to an inward curvature of the HDP, but centrifugal force pulled the atoms back. Snapshots in (e) illustrate that the system could collapse if *ω* was too large.

When the input rotational frequency is low, e.g., *ω* = 20 GHz, the VPE remained unchanged soon after 2 ns, which meant that the shape of the HDP changed slightly during rotation (Figure 2a). The reason was that the two hydrogenated parts on the HDP, i.e., the areas with internal carbon–hydrogen (C–H) bonds, were attached via the strong van der Waals (vdW) force, and the centrifugal force was not high enough to separate them. However, once *ω* reached 30 GHz, the centrifugal force on the HDP was strong enough to overcome the vdW force and separate the C–H areas apart. Simultaneously, HDP was undertaking breathing vibrations (Figure 2). This was the reason why the VPE curves kept fluctuating. 

However, the fluctuation amplitudes did not increase monotonously with the input rotational frequency due to the complex loads on the HDP. For example, for the system with *ω* in the interval of 30–40 GHz, the VPEs varied with small amplitudes (Figure 2b). This was because the centrifugal force and vdW force on the HDPs were not in equilibrium. However, when *ω* = 50 GHz, there was no fluctuation of the VPE curve after 2 ns, which implied the configuration of HDP remained unchanged (Figure 2c). In this case, the centrifugal and vdW forces reached an equilibrium state. When *ω* = 60 GHz, the VPE curve started to fluctuate again. Furthermore, it was evident that when the *ω* was higher than 60 GHz, i.e., 70, 80, and 90 GHz, the VPE curves could oscillate with larger amplitudes (Figure 2d). For example, when *ω* = 90 GHz, the value of VPE oscillated within the range of 5.91–16.94 eV. For the systems with *ω* in the range of 70–90 GHz, the centrifugal force on the HDP was high enough but the vdW force could not act stably anymore, and the HDPs rotated eccentrically and deformed along the z-axis simultaneously. This led to the large deformation of the HDP and obvious fluctuations of the VPE. In addition, when the input rotational speed reached the critical value, i.e., 100 GHz, the VPE approached ≈ 90 eV soon after 1.4 ns. It is known that the centrifugal force is proportional to the square of *ω*. Hence, when *ω* = 100 GHz, the centrifugal force on the HDP was so strong that it pulled the HDP away from the z-axis and further stretched the R-rotor out of the R-stators. In other words, the system collapsed because of the drastic deformation of the HDP (Figure 2e). 

In Figure 1b, due to more atoms on the HDP moving away from the z-axis under the centrifugal force of rotation, the moment of inertia (MoI) curves reached higher mean values under higher rotational frequencies. Moreover, the inertia curves usually fluctuated around their average value, which indicates that the HDP shrunk along the z-axis under most conditions. In particular, the results of the inertia for *ω* ≥ 70 GHz changed periodically, accompanied with large amplitudes. Hence, one can conclude that high rotational speeds led to high centrifugal forces and further led to great deformation of the HDP.

Deformation of the HDP led to a variation of the VPE and MoI. However, this does not mean that the eccentricity (*e*) of the HDP’s centroid varied simultaneously. If the whole rotor, i.e., the L-rotor, R-rotor, and the HDP, had an ideal symmetric deformation about the z-axis, then the value of *e* should be zero. If the HDP had a stable configuration and a constant eccentricity, then the value of *e* should be a positive constant. However, random thermal vibration of the atom always exists during the rotation of the HDP. Hence, even if the rotating part looked symmetric about the z-axis, thermal vibration still led to the eccentricity of the HDP (Figure 1c). The value of *e* at *ω* = 20 GHz, averaged over time, was even bigger than that at 30 GHz due to the special irregular “∞-shaped” configuration of the HDP under a low rotation speed (Figure 2a). When *ω* reached 30 GHz or higher, the up and down layers of the HDP were no longer attached and deformed almost symmetrically. Hence, a lower eccentricity appears. The lowest value of *e* happened at *ω* = 50 GHz because the shape of the deformed HDP was highly symmetrical under the equilibrium centrifugal force and vdW force (Figure 2c). However, as *ω* approached 60 GHz or higher, the stable mean values of *e* significantly increased but were lower than 0.25 Å. At 100 GHz, after 1.4 ns of rotating under extremely high centrifugal forces, the value of *e* exceeded 20 Å or even higher, which indicates that the HDP moved far away from the z-axis, which then resulted in the failure of the whole system.

In our model, the R-rotor connected with the HDP directly. Therefore, when the HDP deformed along the z-axis, the R-rotor was moving correspondingly. Therefore, the distance between the right edges of the R-rotor and R-stator2, i.e., *d*, changed simultaneously. Figure 1d1 lists the history curves of *d* during rotation with the HDP under different input conditions. It was found that the mean value of *d* decreased monotonously with increasing input rotation frequency. Meanwhile, there were some other novel characteristics. For instance, at *ω* = 20 GHz, the fluctuation of *d* could be neglected after no more than 2 ns, i.e., the amplitude was near zero. This implies that the right edge of the R-rotor had no translation during rotation, which demonstrates that the configuration of the HDP remained identical when the centrifugal force was low but the vdW force was high. 

**Figure 3:**
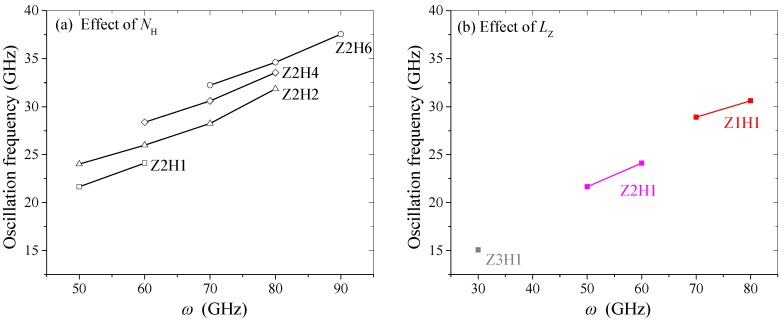
Oscillation frequencies of the *d* curves with amplitudes higher than 0.5 nm during 9–10 ns obtained using a fast Fourier transmission (FFT) method: (**a**) effect of the hydrogenation scheme (*N*_H_) on the HDP and (**b**) effect of the axial length of the HDP (*L*z).

Once *ω* ≥ 30 GHz, the centrifugal force was high enough that the fluctuation of *d* still existed even after reaching a stable state. If *d* fluctuated with a large and stable amplitude, a nanodevice, e.g., an oscillator, could be made from the present model under such conditions. In Figure 1d1,d2, the amplitudes of the *d* curves were 0.18, 0.10, 0.00, and 0.16 nm at 30, 40, 50, and 60 GHz, respectively. However, at 70, 80, and 90 GHz, the amplitudes became 0.51, 0.57, and 0.58 nm, respectively. More importantly, the oscillation amplitudes did not decrease during the simulation. Hence, the oscillations were highly sustainable and reliable. From Figure 2d, one can see that *d* periodically fluctuated between 3.53 nm and 4.70 nm when *ω* = 90 GHz (See Video S1 in the Supplementary Materials). Furthermore, from Figure 3a, one can see that the oscillation frequencies of the *d* curves for a Z2H6 system with an amplitude over 0.5 nm increased with *ω*, e.g., they were 32.23, 34.62, and 37.56 GHz at *ω* = 70, 80, and 90 GHz, respectively. At 100 GHz, the *d* curves became negative after 1.4 ns, which means the R-rotor escaped from the R-stator2, and the system collapsed (See Video S2 in the Supplementary Materials).

### 2.2. Axial Translational Motion in the Z2H6 System at 25 K, 50 K, and 150 K

**Figure 4:**
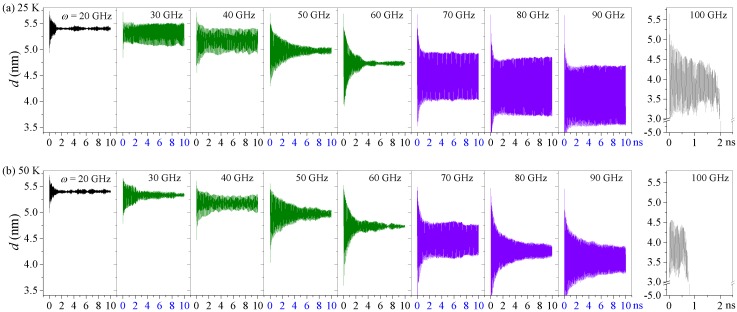

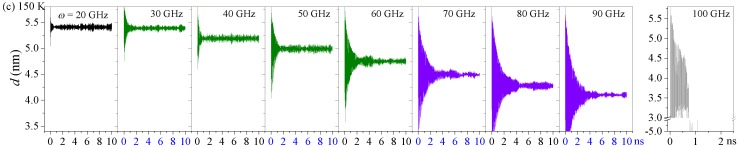
Axial motion of the R-rotor in the Z2H6 system with different inputs at temperatures higher than 8 K: (**a**) at 25 K, (**b**) at 50 K, and (**c**) at 150 K.

For a nanosystem in a canonical NVT ensemble, temperature significantly affects its dynamic response. At higher temperatures, the atoms in the present system had more drastic thermal vibrations, which influenced the interaction (friction) between the stators and rotors. Here, we investigated the axial motions of the R-rotor in the Z2H6 system at temperatures higher than 8 K, i.e., 25 K, 50 K, and 150 K (Figure 4). Compared with the *d* curves at 8 K shown in Figure 1d, we found that the *d* curves at 25 K differed slightly from these curves at 8 K, and the curves of *d* still fluctuated orderly with considerable amplitudes at high rotational frequencies, e.g., *ω* = 70, 80, and 90 GHz. However, when the environment temperature increased to 50 K, the amplitudes of the *d* curves decreased continually with increasing *ω*. In addition, as the temperature approached 150 K, the amplitudes of the *d* curves dampened quickly and reduced to nearly zero within 4 ns. Furthermore, the Z2H6 system at any temperature always collapsed at 100 GHz.

**Figure 5:**
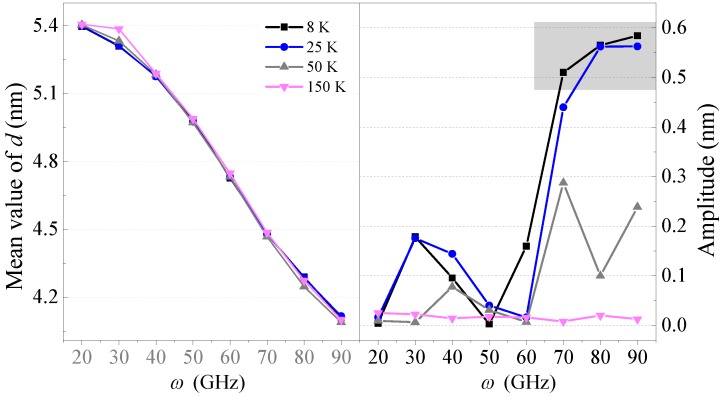
Mean values and amplitudes of *d* curves of the Z2H6 system between 9 ns and 10 ns at different temperatures.

From the left part of Figure 5, one can see that the mean values of *d* at 8 K, 25 K, 50 K, or even 150 K always decreased with the increasing *ω*, and there were no obvious differences among them. This means that temperature had a slight influence on the mean value of *d*. However, in the right part of Figure 5, the amplitude curves of *d* varied significantly with temperature when *ω* was high. The amplitude curve of *d* under 8 K was the highest among the four temperature cases. At 25 K, the amplitude of *d* had a small decrease but was still higher than 0.5 nm at *ω* = 80 GHz and 90 GHz. After that, when the temperature was higher, e.g., 50 K, the amplitudes dropped to 0.29, 0.10, and 0.24 nm at 70, 80, and 90 GHz, respectively. Finally, when the temperature approached 150 K, the amplitudes under all the input rotation frequencies were nearly zero, which indicates that the *d* curves had neither oscillations nor fluctuations. Hence, temperature influenced the amplitude of the *d* curves.

From the above, we concluded that the equilibrium positions of *d* were not influenced by the environmental temperature but the amplitudes of the *d* curves, i.e., the oscillation of R-rotor, were very sensitive to the temperature. Hence, to obtain an oscillator with a high amplitude from the present model, the temperature should be confined to under 50 K.

### 2.3. Effect of the Hydrogenation Scheme (N_H_) on the Axial Oscillation of the R-Rotor at 8 K

In a previous simulation, *N*_H_ = 6, i.e., there were six lines of carbon atoms in each horizontal side of the deform part on the rotor, and they were bonded by hydrogen atoms. Due to the existence of the local C–H bonds, the horizontal sides became curved, which was verified by the simulations above. If we change the value of *N*_H_, what will happen to the output of the system? To demonstrate the effect of *N*_H_, we subsequently reduced *N*_H_ from 6 to 4, 2, and 1.

**Figure 6:**
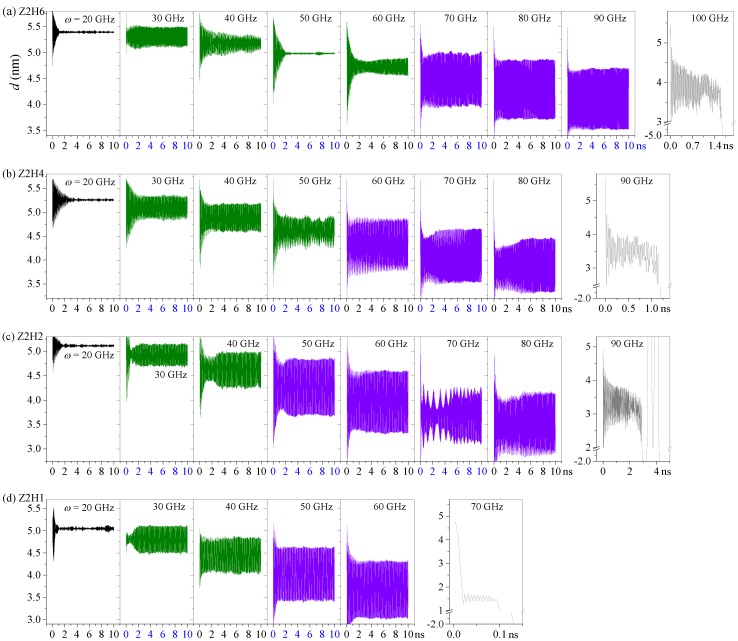
Axial motion (*d*) of the R-rotor with different hydrogenation schemes on the HDP with different rotational frequencies at 8 K: (**a**) Z2H6, (**b**) Z2H4, (**c**) Z2H2, and (**d**) Z2H1. In the Z2H2 system with *ω* = 70 GHz, the “beat” phenomenon happened in the first 5 ns.

**Figure 7:**
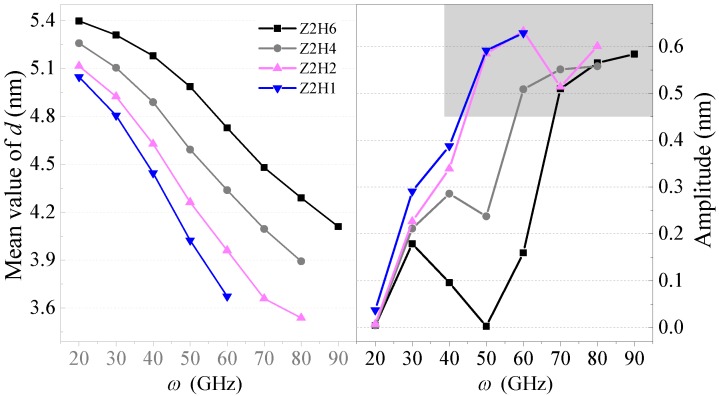
Mean values and amplitudes of the *d* curves in the systems with different hydrogenation schemes at 8 K between 9 ns and 10 ns.

According to the results in Figure 6 and Figure 7, one can see that the output of the system depended on the hydrogenation schemes. Moreover, the equilibrium positions of *d* always decreased as the input rotation frequency increased. The reason was that a larger centrifugal force led to further deformation of the HDP with a higher rotational speed. Furthermore, at the same input rotational frequency, a smaller *N*_H_ induced a lower value of *d*. There were narrower areas on the horizontal sides that remained inwardly concave when *N*_H_ was smaller. Hence, the HDP deformed easier under the same centrifugal force. Furthermore, the critical rotation speed for the schemes of Z2H6, Z2H4, Z2H2, and Z2H1 were 100, 90, 90, and 70 GHz, respectively. Hence, the system with a lower value of *N*_H_ collapsed at a lower rotational frequency. 

It was evident that the system with *ω* > 20 GHz could always produce a valuable oscillation no matter the C–H scheme adopted. By comparing the amplitudes of the *d* curves in the right part of Figure 7, one can see that for the HDP, the amplitude of *d* increased rapidly with *ω*. If *ω* was near the critical value over which the system collapsed, the oscillation amplitudes were higher than 0.5 nm (within the gray background in Figure 7). Furthermore, the largest amplitude of the schemes of Z2H6, Z2H4, Z2H2, and Z2H1 were 0.58, 0.56, 0.63, and 0.63 nm, respectively. Hence, the system could act as a nano-oscillator if *N*_H_ > 0. Furthermore, the value of *N*_H_ did not influence the largest amplitude of the oscillations significantly. If we need a rotor with the HDP to generate a high amplitude with a relatively low input rotation speed, we can choose a lower *N*_H_.

Furthermore, the oscillation frequencies of the *d* curves with amplitudes greater than 0.5 nm for different C–H schemes are shown in Figure 3a. This figure demonstrates that the oscillation frequencies increased linearly with the rotation speed for a specific hydrogenation scheme. One can also see that the oscillation frequency was higher when the system had more lines of C–H bonds on the horizontal sides of the HDP, which implies that an oscillator with a certain period could be obtained by choosing a proper HDP. 

**Figure 8:**
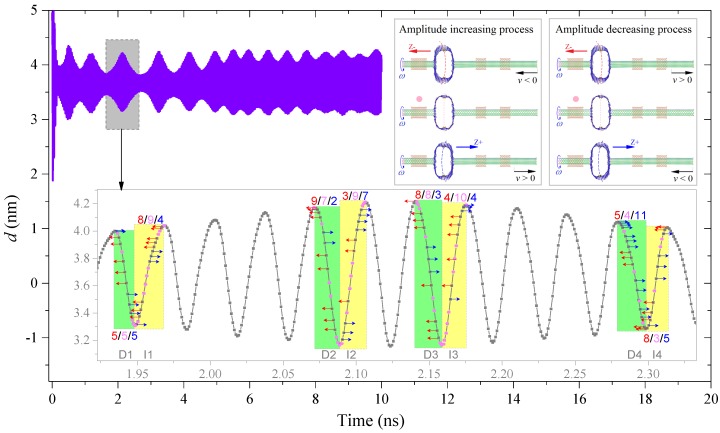
“Beat” phenomenon in the *d* curves of the Z2H2 system with *ω* = 70 GHz at 8 K.

In the above analysis, we also found a “beat” phenomenon in the Z2H2 system when *ω* = 70 GHz at 8 K. By observing the trace of the system, we found that the HDP had both radial deformation and axial bending of the vertical sides of the HDP along the z-axis during the rotation with the rotor. We conclude that the “beat” phenomenon was mainly caused by the axial bending leftward (z−; red arrow) and rightward (z+; blue arrow) alternatively during oscillating. According to the snapshots inserted in Figure 8, when the bending direction of the vertical sides of the HDP was aligned with *v*, i.e., the velocity direction of the right end of the R-rotor, the amplitude of the *d* curves increased. Otherwise, the amplitude decreased. For example, between 1.92 ns and 2.33 ns, the amplitude increased first and then decreased.

The detailed variation processes were as follows. In the increasing stage: when *d* decreased (*v* < 0), there were five “z−” and five “z+” states in the D1 period (Figure 8), and there were nine “z−” and two “z+” states in the D2 period; when *d* increased (*v* > 0), there were eight “z−” and four “z+” states in the I1 period, and there were three “z−” and seven “z+” states in the I2 period. It seems there were more states of the bending direction of vertical sides of the HDP align with the direction of R-rotor; hence, the amplitude of *d* gradually increased. On the contrary, during the decreasing interval: from the D3 to the D4 period, when *d* decreased, the number of “z+” states increased from three to eleven; from the I3 to the I4 period, the number of “z−” states increased from four to eight. Hence, the amplitude of *d* reduced due to more opposite bending states of the HDP to the motion of the R-rotor.

### 2.4. Effect of the Length of the HDP with N_H_ = 1 on Its Oscillation at 8 K

**Figure 9:**
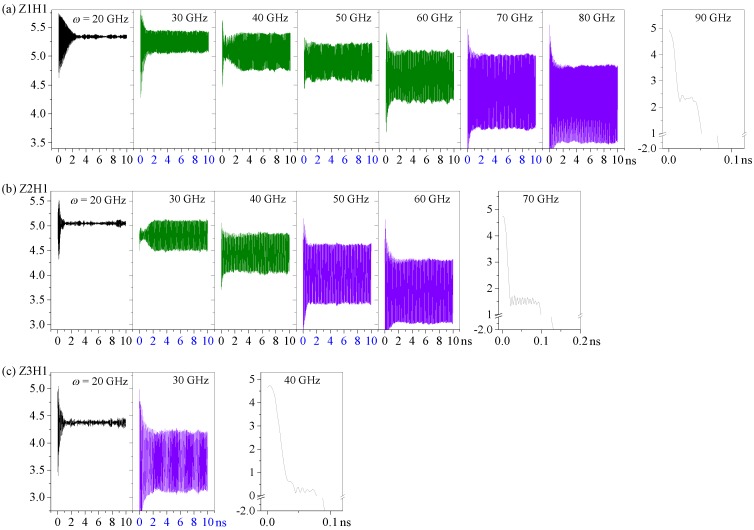
Axial motions of the R-rotor connected with the HDPs with different lengths at 8 K: (**a**) Z1H1 HDP, (**b**) Z2H1 HDP, and (**c**) Z3H1 HDP.

**Figure 10:**
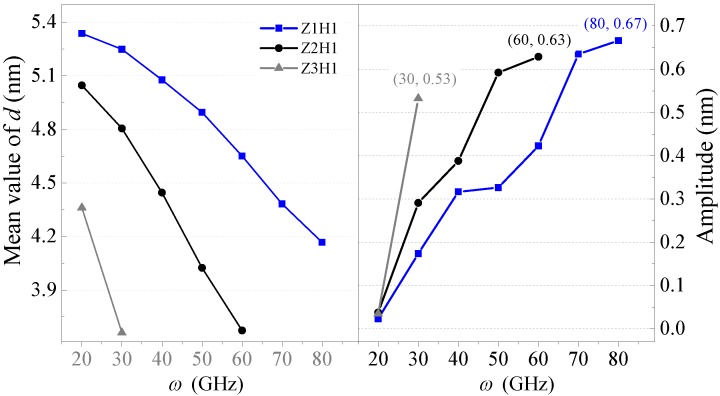
Equilibrium positions (mean values of *d*) and oscillation amplitudes of the R-rotor connected with the HDPs that had different lengths at 8 K between 9 ns and 10 ns.

Figure 9 and Figure 10 show the axial movement of the R-rotor connected with the HDPs with different lengths (*L*z). From Figure 9, as the length of the HDP in the z-axis increased, the critical rotational frequency dropped quickly. For example, the systems with Z1H1, Z2H1, and Z3H1 HDPs collapsed at 90 GHz, 70 GHz, and 40 GHz respectively. This was because the longest HDP had the lowest stiffness but the largest inertia and the largest centrifugal force when the rotation frequency was specified. Therefore, its shape changed the easiest and it had the largest deformation. For example, when *ω* increased from 20 to 30 GHz, the value of *d* reduced to 0.09 nm and 0.24 nm for the Z1H1 and Z2H1 systems, respectively. For the Z3H1 system, the reduction was even larger, i.e., over 0.70 nm. Before collapsing, the system could always reach its dynamically stable state within 6 ns, and had regular oscillations when *ω* was higher than 20 GHz.

From the amplitudes of the *d* curves shown in Figure 10, one can see that the oscillation amplitude increased with *ω*, and the amplitude was higher when HDP was longer if *ω* was specified. The output oscillations of the R-rotor with Z1H1, Z2H1, and Z3H1 HDPs had maximum amplitudes of 0.67, 0.63, and 0.53 nm when *ω* = 80, 60, and 30 GHz, respectively. For a system requiring a larger amplitude oscillation or a larger decrease of *d* with a lower rotation frequency, we can choose a longer HDP. 

Furthermore, the statistical results of the oscillation frequencies in the *d* curves with amplitudes larger than 0.5 nm are given in Figure 3b. One can conclude that the oscillation frequencies increased as the length of the HDP decreased. For example, the oscillation frequency for the Z3H1 system at *ω* = 30 GHz was only ≈ 15 GHz. For the Z2H1 system, the oscillation frequencies were between 21 GHz and 24 GHz. For the Z1H1 system, the oscillation frequency of the R-rotor jumped to 29–31 GHz. Hence, by changing the length of the HDP, the system could produce axial oscillation with a wide range of frequencies.

## 3. Materials and Methods 

### 3.1. Model 

**Figure 11:**
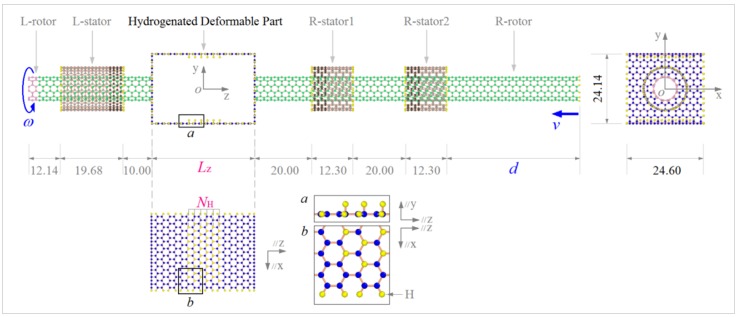
Initial schematic of a rotation–translation nanoconvertor with a hydrogenated deformable part (HDP) on the rotor from carbon nanotube (CNT) (6, 6). *ω* is the input rotational frequency exerted on the left edge of the L-rotor. *d*, the output of the system, is the axial distance between the right edges of the R-rotor and the R-stator2 from CNT (11, 11). The right edge of the R-rotor has a translational velocity of *v* during the deformation of the HDP. On the internal sides of the up and down layers of the HDP, the *N*_H_ lines of neighboring carbon atoms were hydrogenated. *N*_H_ was no more than six. The rotors were made from a (6, 6) CNT, and stators from a (11, 11) CNT. The dimension unit is Å. “//z” means parallel to z-axis.

In this work, the model shown in Figure 11 was adopted to design a nanoconvertor for translating rotation into motion. When a rotational speed, i.e., *ω*, is exerted on the left end of a rotor, the HDP deforms under a centrifugal force and moves away from the rotational axis of the rotor. Simultaneously, asymmetric van der Waals (vdW) forces on both surfaces of the HDP lead to a reverse deformation. When the two deformations reach a dynamic equilibrium state, the value of *d* will have a stable fluctuation, which can be used to design a perfect nano-oscillator. For a deep understanding of the dynamic oscillation behavior of the R-rotor during rotation, more factors, including temperature, hydrogenation schemes (*N*_H_), and the length of HDP (*L*z), were investigated using molecular dynamics simulations. Six HDP models with different values of *N*_H_ and *L*z were considered. Except for the HDP, the sizes of the remaining components in the model remained unchanged. Furthermore, each carbon atom on the boundary of HDP and the ends of all three stators and the R-rotor was bonded with a hydrogen atom. The detailed parameters of the HDPs are listed in Table 1. 

### 3.2. Methodology

#### 3.2.1. Features of the HDP

To reflect the state of the HDP, its potential energy was recorded. In particular, the variation of the potential energy (VPE) illustrates the change of its state. The concept of the VPE of a HDP can be calculated by subtracting the current potential energy from its initial value, i.e.,

VPE(t) = PE(t) − PE(t_0_), (1)

where PE(t) and PE(t_0_) are the potential energy of the HDP at time t and t_0_ (<t), respectively. The value of PE(t) can be obtained by substituting the positions of atoms in the component at time t into a potential function, e.g., the adaptive intermolecular reactive empirical bond order (AIREBO) potential [45].

The moment of inertia (MoI) of an HDP can be defined as:

(2) $$\mathrm{MoI}(t)=\sum_{i=1}^{M}m_{i}{\left(x_{i}^{2}+y_{i}^{2}\right)}_{t},$$

where *x_i_*, *y_i_*, and *m_i_* are the coordinates and mass of the *i*th atom in the HDP with a total of *M* atoms. For a deformed HDP, the value of MoI depends on two factors, i.e., the eccentricity (*e*) and the deformation of the component. If the component has a small eccentricity, the degree of deformation of the HDP can be illustrated by the variation of the MoI.

The eccentricity of the deformed HDP can be defined as:

(3) $$e\left(t\right)=\sqrt{{\left(\sum_{i=1}^{M}\frac{m_{i}x_{i}}{m_{i}}\right)}_{t}^{2}+{\left(\sum_{i=1}^{M}\frac{m_{i}y_{i}}{m_{i}}\right)}_{t}^{2}}.$$

The centrifugal force on a HDP can be expressed as:

(4) $${\vec{F}}_{C}=\sum_{i=1}^{M}{\vec{F}}_{Ci}=\sum_{i=1}^{M}m_{i}\vec{\omega }\times \left(\vec{\omega }\times \vec{r_{i}}\right),$$

where ${\vec{F}}_{Ci}$ is the centrifugal force applied on atom *i* in the HDP, and $\vec{r_{i}}$ is the vector from atom *i* to “*o*”, i.e., the origin of reference in coordinates xyz. The direction of ${\vec{F}}_{Ci}$ is normal to the z-axis.

#### 3.2.2. Flowchart of the Molecular Dynamics Simulation

The molecular dynamics simulation approach was adopted to investigate the deformation behavior of the rotor during rotation. The simulations were carried out on the large-scale atomic/molecular massively parallel simulator (LAMMPS) [46]. In this study, considering electric neutrality, the interaction between the carbon and/or hydrogen atoms was evaluated using the AIREBO potential, which contains three items, i.e., item one is the reactive empirical bond order (REBO) part for describing the bond-ordered short-range interaction, item two is the torsion part to consider the dihedral effect in the local deformation, and the final item is the Lennard–Jones potential (L-J) [47] to describe the vdW interaction between two atoms with a distance smaller than 1.02 nm. The force can be expressed as:

(5) $$F_{\mathrm{vdW}}=\left|\partial U_{L-J}/\partial r_{ij}\right|,$$

where U_L-J_ is the L-J potential energy and *r_ij_* is the distance between atoms *i* and *j*.

For each simulation, it contained the following five major steps:

Step 1: Create the model of the nanoconvertor with a specific HDP.

Step 2: Reshape the initial configuration of the model by minimizing its potential energy with the steepest descent method.

Step 3: Fix the three rings of atoms on the left edge of the L-rotor and four rings of atoms on each stator; then, relax the rest of the system in a canonical (NVT) ensemble using a Nose–Hover thermostat at a given temperature for 100 ps. The time step was 0.0005 ps.

Step 4: Provide a specified rotational frequency on the left end of the L-rotor, which was previously fixed. The time step was modified to be 1 fs.

Step 5: record the necessary physical quantities of HDP including VPE, MoI, *e*, and *d* for postprocessing.

#### 3.2.3. Fast Fourier Transmission

The output curves of *d* may have obvious fluctuations during the rotation of the system at finite temperatures. To accurately describe the vibration, the fluctuation frequencies of the *d*-t curves were analyzed using the fast Fourier transmission (FFT) approach [48,49] using the following equation:

(6) $$d(t)=\sum_{i=1}^{I}A_{i}\sin\left(2\pi \cdot \Omega_{i}\cdot t+\phi_{i}\right),$$

where $A_{i}$ is the *i*th order of amplitude, $\Omega_{i}$ is the eigenfrequency, and $\phi_{i}$ is the corresponding phase angle. In this study, all the transmissions were operated on the last 1000 ps of the curves.

## 4. Conclusions

Using a molecular dynamics simulation approach, we investigated the dynamic response of a rotation–translation nanoconvertor, which contained a rotor and three stators. The rotor had three parts, i.e., the L-rotor and R-rotor for inputting rotation (*ω*) and outputting axial motion (*d*), respectively, and the two parts were connected by an internally hydrogenated deformed part (HDP). After a series of numerical tests of the physical quantities of the HDP and the axial output of the R-rotor at a low temperature, the following five conclusions were drawn. 

First, at extremely low temperatures, e.g., 8 K, the configuration of HDP remained unchanged with a low rotational speed due to the two hydrogenated parts on the HDP curve that were attached together via a strong van der Waals force. Once the centrifugal force was strong enough to overcome the van der Waals force, HDP displayed a breathing variation and the axial motion of the R-rotor occurred simultaneously. In particular, when *ω* was high enough, the R-rotor could oscillate with an amplitude over 0.5 nm in the present model. This characteristic can be applied to the design of an oscillator. 

Second, the mean value of the historical curve of *d* with respect to a specified *ω* depended slightly on the environmental temperature. However, its amplitude varied significantly with temperature, especially when *ω* was high. To obtain an oscillator with a high amplitude from the present model, the temperature should be lower than 50 K.

Third, the hydrogenation scheme on the HDP (i.e., the value of *N*_H_) had a significant influence on the output of the system. At a specific temperature, the mean value of *d* always decreased with increasing of *ω*. If *ω* was fixed, a smaller *N*_H_ induced a lower mean value of *d*. Furthermore, the system could always generate large-amplitude oscillations when *N*_H_ > 0. Moreover, the “beat” phenomenon was found and its mechanism was revealed. 

Fourth, the length of the HDP along z-axis (*L*z) also had a significant effect on the dynamic behavior of the system. A system with a longer HDP could produce an oscillation with a larger amplitude, even at a lower rotation frequency.

Final, *ω*, *N*_H_, and *L*z all influenced the oscillation frequency of the R-rotor, i.e., the oscillation frequency could be adjusted in a wide range by changing the input rotational speed of the rotor and/or hydrogenation scheme and/or the length of the HDP.

For the potential fabrication of our system, some essential techniques are demonstrated below: 

First, create a double-walled CNTs with a perfect structure and certain chirality using one of a range of methods, such as conventional arc-plasma methods or chemical vapor deposition. 

Second, create the major parts of the system, stators, and tubes on the rotor, e.g., by electrical-breakdown technique.

Third, form an HDP by hydrogenating the inside of a CNT with a large radius; potential methods that could be used include chemical vapor deposition, chemisorption, and an electrical-breakdown technique. 

Finally, merge the HDP on the rotor to form a system, as shown in Figure 11. 

## Figures and Tables

**Table 1:** Initial parameters of the HDPs in the nanoconvertor in Figure 11.

Model		*L*_Z_/Å	*N*_H_	Number of Atoms on HDP
HDP	Z1H1	26.99	1	960C + 96H (boundary) + 16H (*N*_H_)
	Z2H1	35.51	1	1128C + 112H (boundary) + 16H (*N*_H_)
	Z2H2	35.51	2	1128C + 112H (boundary) + 34H (*N*_H_)
	Z2H4	35.51	4	1128C + 112H (boundary) + 68H (*N*_H_)
	Z2H6	35.51	6	1128C + 112H (boundary) + 102H (*N*_H_)
	Z3H1	48.29	1	1380C + 136H (boundary) + 16H (*N*_H_)

## Supplementary Materials

The following are available online at https://www.mdpi.com/1424-8220/20/7/1969/s1, Video S1: Z2H6-T = 8 K- *ω* = 90 GHz-during [9.9, 10] ns.avi; Video S2: Z2H6-T = 8 K- *ω* = 100 GHz-during [1.46, 1.66] ns.avi.
